# Patterns of *MHC-G-Like* and *MHC-B* Diversification in New World Monkeys

**DOI:** 10.1371/journal.pone.0131343

**Published:** 2015-06-29

**Authors:** Juan S. Lugo, Luis F. Cadavid

**Affiliations:** Department of Biology and Institute of Genetics, Universidad Nacional de Colombia, Bogotá, Colombia; National Cheng-Kung University, TAIWAN

## Abstract

The MHC class I (MHC-I) region in New World monkeys (Platyrrhini) has remained relatively understudied. To evaluate the diversification patterns and transcription behavior of MHC-I in Platyrrhini, we first analyzed public genomic sequences from the *MHC-G-like* subregion in *Saimiri boliviensis*, *Ateles geoffroyi* and *Callicebus moloch*, and from the *MHC-B* subregion in *Saimiri boliviensis*. While *S*. *boliviensis* showed multiple copies of both *MHC-G-like* (10) and *–B* (15) loci, *A*. *geoffroyi* and *C*. *moloch* had only three and four *MHC-G-like* genes, respectively, indicating that not all Platyrrhini species have expanded their MHC-I loci. We then sequenced *MHC-G-like* and *-B* cDNAs from nine Platyrrhini species, recovering two to five unique cDNAs per individual for both loci classes. In two *Saguinus* species, however, no *MHC-B* cDNAs were found. In phylogenetic trees, *MHC-G-like* cDNAs formed genus-specific clusters whereas the *MHC-B* cDNAs grouped by Platyrrhini families, suggesting a more rapid diversification of the former. Furthermore, cDNA sequencing in 12 capuchin monkeys showed that they transcribe at least four *MHC-G-like* and five *MHC-B* polymorphic genes, showing haplotypic diversity for gene copy number and signatures of positive natural selection at the peptide binding region. Finally, a quantitative index for MHC:KIR affinity was proposed and tested to predict putative interacting pairs. Altogether, our data indicate that *i)* MHC-I genes has expanded differentially among Platyrrhini species, *ii) Callitrichinae* (tamarins and marmosets) *MHC-B* loci have limited or tissue-specific expression, *iii) MHC-G-like* genes have diversified more rapidly than *MHC-B* genes, and *iv)* the MHC-I diversity is generated mainly by genetic polymorphism and gene copy number variation, likely promoted by natural selection for ligand binding.

## Introduction

Major Histocompatibility Complex (MHC) class I molecules present processed peptides to CD8+ T lymphocytes and interact with Natural Killer (NK) cell receptors to modulate immune responses directed against self-altered cells [1]. The hallmark of the MHC class I system is the high genetic polymorphism of its loci, which has been promoted by selective pressures from rapidly evolving pathogens to evade immune recognition [2]. In humans, there are six functional MHC class I genes, *HLA-A*, -*B*. -*C*, -*E*, -*F*, and -*G*, that, together with 11 MHC class I pseudogenes and several non-MHC genes and pseudogenes, constitute MHC class I region on chromosome 6p21 [3]. *HLA-A*, -*B*, and -*C* genes are known as classical MHC class I genes and are characterized by their high polymorphism and expression on virtually all nucleated cells, whereas *HLA-E*, -*F*, and -*G* are nonclassical MHC class I genes displaying limited polymorphism and restricted tissue distribution [4]. The human MHC class I region is divided into three contiguous subgenomic sections, often referred to as the alpha block, located at the telomeric end of the region and containing *HLA-F*, -*G*, and -*A*, the beta block, located at the centromeric end of the region and containing *HLA-B* and -*C*, and the kappa block, at the central part of the region, containing *HLA-E* [5].

Functional MHC class I loci have remained relatively well conserved in Catarrhini primates (humans, apes and Old World monkeys) [6]. Species from the *Hominidae* family (humans and great apes) have orthologous genes to *HLA-A*, -*B*, -*C*, -*E*. -*F*, and -*G*, although *MHC-C* is present in only about 50% of orangutan (*Pongo pygmeus)* haplotypes [7]. The *Hylobatidae* family (gibbons or lesser apes) has at least three *MHC-B* genes and a single copy of each *MHC-A*, -*E*, and -*F* genes, but they lack *MHC-C* and *MHC-G* loci [8]. Likewise, Old World monkeys (family *Cercopithecidae*) have multiple copies of the *MHC-A* and-*B* loci, lack *MHC-C*, have maintained *MHC-E and —F* as single copy genes, and *MHC-G* is a pseudogene [9]. Platyrrhini species (New World monkeys) are distributed from Mexico to the North of Argentina and shared a common ancestor with Catarrhini some 35–45 million years ago (MYA) [10]. At least 15 Platyrrhini genera have been recognized, which are grouped into three families: *Pitheciidae*, *Atelidae*, and *Cebidae* [11]. Platyrrhini are typically known to express classical MHC class I loci more closely related to *MHC-G* [12, 13], lacking orthologues of *MHC-A* and *MHC-C* loci, and containing a functional *MHC-E* [14]. A genomic analysis of the MHC class I alpha block (*MHC-F/G/A*) in the Platyrrhini species *Callithrix jacchus* (common marmoset) revealed the presence of five *MHC-G-like* genes, nine *MHC-G-like* pseudogenes, one *MHC-F* gene, and four *MHC-F* pseudogenes [15]. The expansion of this region in the common marmoset has probably occurred by repeated segmental duplications of units containing four *MHC-G-like* and one *MHC-F* genes [15]. In addition, genomic sequencing of the MHC class I beta block (MHC-*B/C*) in this species showed the presence of nine *bona fide MHC-B* genes, five of which were putatively transcribed [16]. However, cDNA sequencing efforts to identify *MHC-B* transcripts in this species have given mixed results. In a population of 15 common marmosets, van der Wiel et al (2013) failed to identify any *MHC-B* transcript [17], although Cao et al (2015) found four unique *MHC-B* sequences with one to three sequences per individual in a population of eight common marmosets [16]. This suggests that *MHC-B* loci have either low transcription levels that hinder their detection by conventional PCR-based techniques, and/or they have differential tissue expression. Although the vast majority of classical MHC class I cDNAs reported in Platyrrhini are from the *MHC-G-like* loci, cDNAs with high similarity to *MHC-B* have been identified in the owl monkey (*Aotus* sp) [18], the saki monkey (*Pithecia pithecia*) [13], the white-bellied spider monkey (*Ateles belzebuth)* [13], and the brown-headed spider monkey (*Ateles fusciceps)*. Furthermore, sequence and retroelement analyses of the 5′-flanking regions of MHC class I genes, have provided evidence for the presence of various copies of *MHC-B* in the owl monkey, the capuchin monkey (*Cebus apella*), and the spider monkey [18].

In order to gain a better understanding of the MHC class I genes in Platyrrhini, we have investigated their diversification processes at three different levels, *i*) at the genomic level by analyzing the MHC class I gene organization in three Platyrrhini species using public sequence information, *ii*) at the transcriptomic level by cloning and sequencing *MHC-G-like* and *MHC-B* cDNAs from 10 species representing two Platyrrhini families (*Cebidae* and *Atelidae*), and *iii*) at the gene level by studying the polymorphism of *MHC-G-like* and *MHC-B* loci in a population of 12 white-fronted capuchin monkeys (*Cebus albifrons*).

## Materials and Methods

### Ethics Statement

We used samples from the following primate species: cotton-top tamarin (*Saguinus oedipus*), white-footed tamarin (*Saguinus leucopus*), squirrel monkey (*Saimiri sciureus*), tufted capuchin monkey (*Cebus apella*), white-headed capuchin monkey (*Cebus capucinus*), red howler monkey (*Alouatta seniculus*), white-bellied spider monkey (*Ateles belzebuth*), brown spider monkey (*Ateles hybridus*), and common woolly monkey (*Lagothrix lagotricha*). Animals used to obtain blood samples were maintained at the Center for Rescue and Rehabilitation of Wild Fauna (URRAS) from the Universidad Nacional de Colombia′s Veterinary School. The Center is authorized by the Bogotá Secretary of the Environment following the Colombian legislation on the use of animals for research (law 84/1989, resolution 8430/1993), and it is regulated by the Bioethics Committee of the Universidad Nacional de Colombia′s Veterinary School, directed by Dr. Gloria C. Ramírez-Nieto (gcramirezn@unal.edu.co) (Art. 22, Resolution 138/2011). Colombia’s Ministry of the Environment and Sustainable Development authorized the access to genetic resources for scientific research without commercial interests (contract No. 1 of 2012). The study was approved by the Center’s director, Dr. Claudia Brieva (cibrievar@unal.edu.co), as no Institutional Animal Care and Use Committee has been formally established at the University. For animal care monitoring, the results of the present study were reported to the Center’s Director, which in turn reports periodically to the Bioethics Committee. The monkeys were temporarily maintained in cages of 1 x 1.5 x 2 meters with an average temperature of 22 degrees centigrade, and a relative humidity of 67%. The monkeys’ diet was based on a supply of fruits, vegetables and a nutritional supplement including vitamins, minerals and proteins. Their environment was enriched by including visual barriers (to avoid social conflicts), feeding devices, some branches and vegetation, perches and a nest box. Trained veterinary personnel carried out all procedures requiring the handling of animals. Blood samples were obtained as part of routine animal care by the Center′s veterinarian (Dr. Robin Poches), from animals anesthetized with Ketamine 20 mg/Kg or Tyletamine/Zolazepam (Zoletil) 4.4 mg/Kg. No animals were sacrificed during, or as a result of, this study.

### MHC class I region genomic sequences

BLAST searches against the NCBI GenBank database using known MHC class I sequences were conducted to identify Platyrrhini bacterial artificial chromosomes (BACs) and next generation sequence scaffolds containing MHC class I genes. Significant BLAST matches (identity > 80% and e-value < 10^−70^) were found in the Bolivian squirrel monkey (*Saimiri boliviensis*), the spider monkey (*Ateles geoffroyi*), and the dusky titi monkey (*Callicebus moloch*). Data from the former two species were produced by the National Institutes of Health—Intramural Sequencing Center and released on April 2010. Data from the latter species were produced by the Broad Institute as part of a genome project and released on October 2011. Gene predictions on the genomic sequences were carried out with the GeneScan tool (genes.mit.edu/GENSCAN.html) and with the Augustus software (augustus.gobics.de/), and the identified genes were annotated by BLAST against the NCBI Reference Sequence Database (RefSeq, www.ncbi.nlm.nih.gov/refseq). Genomic sequences were aligned with the Mauve multiple genome alignment software v2.3.1 [19].

### Samples, cDNA cloning, and sequencing

Peripheral blood samples (0,5–1,0 ml) were obtained by femoral venipuncture from a single individual of the following Platyrrhini species: cotton-top tamarin (*Saguinus oedipus*), white-footed tamarin (*Saguinus leucopus*), squirrel monkey (*Saimiri sciureus*), tufted capuchin monkey (*Cebus apella*), white-headed capuchin monkey (*Cebus capucinus*), red howler monkey (*Alouatta seniculus*), white-bellied spider monkey (*Ateles belzebuth*), brown spider monkey (*Ateles hybridus*), and common woolly monkey (*Lagothrix lagotricha*). For polymorphism analyses, 12 unrelated white-fronted capuchin monkeys (*Cebus albifrons*) were also sampled. Total RNA was isolated from peripheral blood with the Trizol reagent (Invitrogen) and used for complementary DNA (cDNA) synthesis with the RevertAid First strand cDNA Synthesis kit (Fermentas). Full-length MHC class I cDNAs were amplified by PCR with generic primers designed for both *MHC-G-like* and *MHC-B*, as follows: forward primer 5'-TAACGGTCMTGGMGCCCCGAA-3' (where M means A or C), and reverse primer 5'-AATGAGAGACACATCAGAGCC-3'. The reaction contained 400 μM dNTPs, 2 mM MgCl2, and 0.5 U of Taq DNA polymerase (Invitrogen) in a volume of 25 μl. Amplifications were carried out with an initial cycle of 5 min at 94°C, followed by 35 cycles of 94°C for 45 s, 62.6°C for 55 s, and 72°C for 65 s, with a final extension of 72°C for 10 min. PCR products were gel-purified with the PureLink kit (Life Technologies), cloned into the pGEM-T Easy vector (Promega), and at least 24 clones per individual were sequenced with the Sanger method at Macrogen (Korea). Reported sequences derived from at least three identical plasmid clones.

### Sequence and evolutionary analysis

Genomic and cDNA sequences were aligned with the Muscle software [20] and the alignment were manually edited using the MEGA v5.2 software [21]. Sequence distances were estimated with the Maximum Composite Likelihood model [22] with standard errors estimated by bootstrap after 1,000 replicates using MEGA v5.2. Phylogenetic analyses were carried out with the Neighbor-Joining, Maximum Likelihood, and Bayesian approaches based on nucleotide substitution models estimated by the ModelTest software v3.7 [23]. Neighbor-joining and Maximum likelihood phylogenies were constructed with the MEGA v5.2 package [21] and their reliability was evaluated by bootstrap with 1,000 replicates. Bayesian phylogenies were inferred with MrBayes v3.2 [24, 25] using 10,000,000 iterations in five chains. Natural selection acting on MHC class I genes was evaluated by codon-based selection analysis using the PAML v4.4 package [26]. For this, the dN / dS ratio (ω) was estimated by maximum likelihood using the F3X4 model for codon frequencies. The neighbor-joining tree generated previously was used to estimate the tree topology likelihood comparing a null model that does not allow ω > 1 (M1) with three models that does (M2, M3, and M8). Comparisons were carried out by likelihood ratio tests and their significance was evaluated by comparing two times the likelihood difference between the models (2ΔL) with a Chi-square distribution with two degrees of freedom. Codons with ω > 1 were identified through a Bayes Empirical Bayes approach [27] when at least two of the three selection models had a probability greater than 0.9.

### Prediction of MHC-I:KIR interactions

Structural models of interacting MHC class I and KIR (killer cell Ig-like receptor) molecules were constructed by homology with the Swiss-Model suite [28] based on the crystallographic structure of human HLA-B*5701 bound to KIR3DL1 (PDB: 3VH8) [29]. Structures were aligned to predict the amount of H-bonds and the desolvation energy between each MHC:KIR pair by using the software Pymol v1.6 (www.pymol.org) and FastContact v2.0 [30], respectively. Potential interacting pairs were selected based on the highest numbers of H-bonds and the lowest desolvation energy by applying the interaction index formula *I = kh / ln dG*, where k is the average energy of an H-bond, h is the number of H-bonds in the pair, and dG is the desolvation energy. The index was tested with experimental data derived from human, chimpanzee, and Rhesus monkey MHC:KIR interactions [29, 31–34] in order to asses its predictability and to quantify the rate of false positives and false negatives.

## Results

### Genomic diversification of the MHC class I region in Platyrrhini

**Fig 1:**
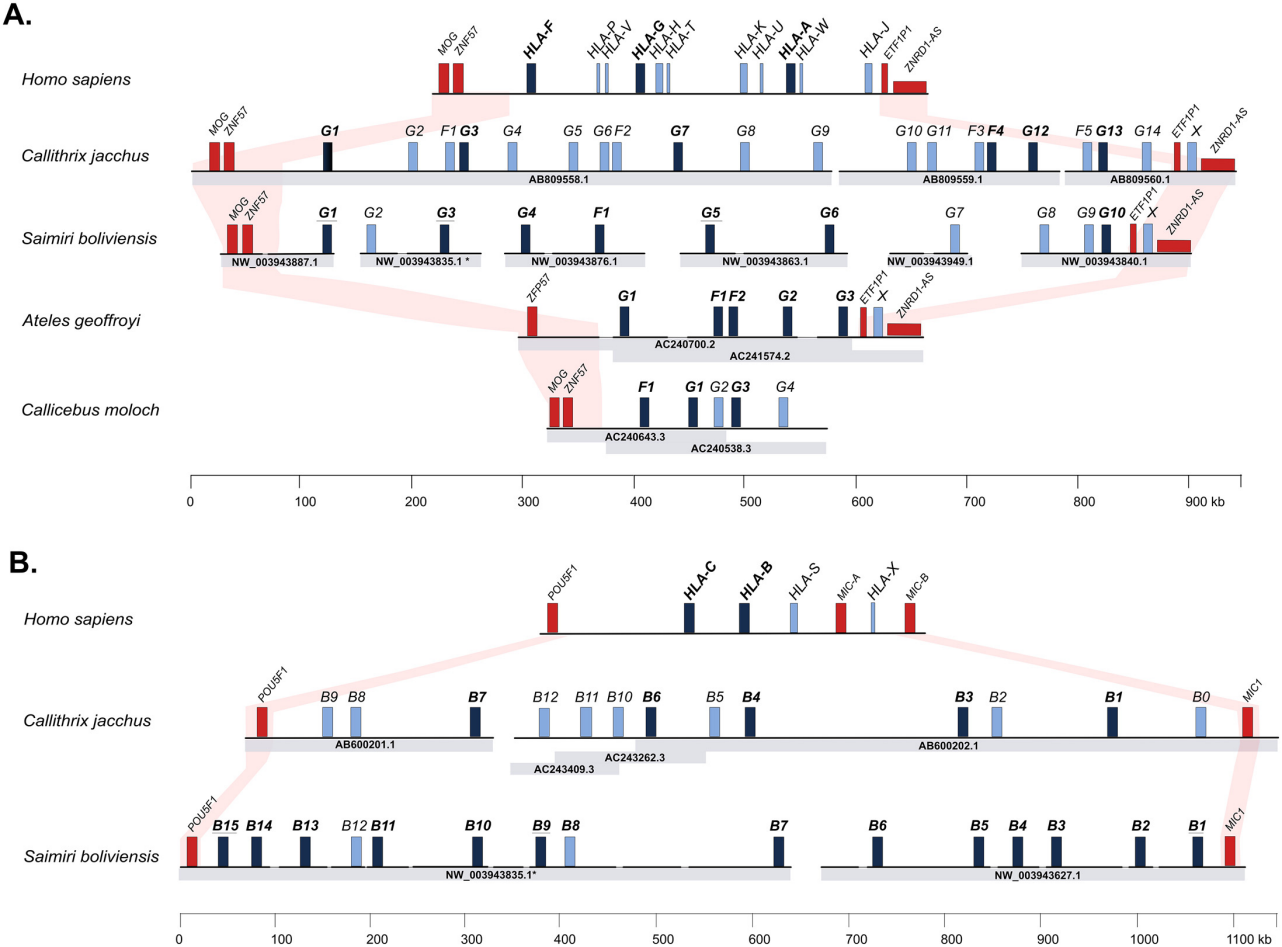
Comparison of the MHC class I region of *S*. *boliviensis*, *A*. *geoffroyi*, and *C*. *moloch* using as reference that of humans [3] and *C*. *jacchus* [15, 16]. A, MHC class I alpha block (*MHC-A/G/F*). B, MHC class I beta block (*MHC-B/C*). Red rectangles represent centromeric and telomeric framework genes, dark blue rectangles indicate putatively transcribed MHC class I genes, including partially sequenced genes (names underlined), and light blue rectangles depict MHC class I pseudogenes. BAC clone or scaffold accession numbers are indicated for each species. The *S*. *boliviensis* scaffold NW_003943835.1 contained *MHC-G-like* and *MHC-B* sequences, likely reflecting an erroneous assembly, but for clarity, the genes were located in their respective regions. Non-MHC class I sequences annotated in these regions are shown in S1 Table.

Sequence database searching using known MHC class I sequences resulted in the identification of BAC clone sequences and next generation sequencing scaffolds containing unpublished MHC class I genes from the Bolivian squirrel monkey (*Saimiri boliviensis*), the spider monkey (*Ateles geoffroyi*), and the dusky titi monkey (*Callicebus moloch*). The search of MHC class I sequences in *S*. *boliviensis* yielded eight non-overlapping next-generation sequence scaffolds, with the first six (NW_003943887.1, NW_003943835.1, NW_003943876.1, NW_003943863.1, NW_003943949.1 and NW_003943840.1) spanning the entire *MHC-F/G/A* region. This subgenomic block was circumscribed at the telomeric end by the framework genes *MOG* (myelin oligodendrocyte glycoprotein) and *ZNF57* (zinc finger protein-57) and at the centromeric end by framework genes *ETF1P1* (eukaryotic peptide chain release factor subunit 1-like) and the non-coding RNA *ZNRD1-AS1* (Fig 1A). The scaffolds contained six *MHC-G-like* genes (five partially sequenced), four *MHC-G-like* pseudogenes, one partially sequenced *MHC-F* gene, and 44 non-MHC genes, pseudogenes, and non-coding open reading frames (ORFs) (Fig 1A and S1 Table). In addition, an MHC class I sequence (tentatively named *MHC-X*) with no close similarity to known primate MHC class I genes was found between the centromeric framework genes *ETF1P1* and *ZNRD1-AS1* (Fig 1A). One of the six scaffolds spanning the *MHC-F/G/A* region (NW_003943840.1) extended beyond the alpha block into the kappa block (*MHC-E*) and contained this block′s telomeric framework gene *RNF39* (gene ring finger protein 39) and seven non-MHC genes also found in humans and in the common marmoset (S1 Fig). The following scaffold (NW_003943826.1) spanned part of the MHC class I kappa block and had a single *MHC-E* gene (*Sabo-E*), together with 12 non-MHC genes and the centromeric framework genes *CDSN* (corneodesmosin), *CCHR1* (coiled-coil alpha-helical rod protein-1), and *TCF19* (transcription factor 19) (S1 Fig). Finally, scaffolds NW_003943835.1 and NW_003943627.1 contained genes and pseudogenes homologous to those of the primate MHC class I beta block (*MHC-B/C*), with the framework gene *POU5F1* (encoding the POU domain, class 5, transcription factor 1) flanking the region at the telomeric end, and a *MIC* gene (MHC class I chain related) at the centromeric end (Fig 1B). These two scaffolds contained 15 *MHC-B* genes, two *MHC-B* pseudogenes (*Sabo-B8* and-*B12*), and 24 non-MHC genes, pseudogenes, and non-coding ORFs (Fig 1 and S1 Table). Scaffold NW_003943835.1 was unusual in that it contained two *MHC-G-like* and nine *MHC-B* sequences, suggesting that the scaffold has been misassembled.

In *A*. *geoffroyi*, two BAC clone draft sequences were identified containing MHC class I sequences. The first BAC clone sequence (AC240700.2) was approximately 218,6 kb long and had seven ordered fragments separated by gaps of unknown size, while the second (AC241574.2) was approximately 225,1 kb and had eight ordered fragments. These two BAC clones overlapped by approximately 170 kb forming a contig of 278.6 kb with six gaps of unknown length (Fig 1A). This contig spanned the entire region homologous to the primate MHC class I alpha block, and was delimited at the telomeric end by the framework gene *ZNF57* and at the centromeric end by the framework genes *ETF1P1* and *ZNRD1-AS1* (Fig 1A). This region had three *MHC-G-like* genes (*Atge-G1*, -*G2*, and -*G3*), two *MHC-F* genes (*Atge-F1* and-*F2*), 25 non-MHC genes, pseudogenes, and non-coding ORFs, and, in addition, an *MHC-X* sequence located between the centromeric framework genes *ETF1P1* and *ZNRD1-AS1* (Fig 1A and S1 Table). Finally, the BLAST search in *C*. *moloch* resulted in two BAC clone sequences (AC240643.3 and AC240538.3) of 161,5 kb and 201,3 kb, respectively, which overlapped by a region of approximately 120 kb, resulting in a contig of approximately 240 kb with no internal gaps (Fig 1A). This region was homologous to the primate MHC class I alpha block, having the telomeric framework genes *MOG* and *ZNF57*, but lacking centromeric framework genes, indicating that the region has not been completely covered. The contig contained two full-length *MHC-G-like* genes (*Camo-G1* and-*G3*), two *MHC-G-like* pseudogenes (*Camo-G2* and-*G4*), one *MHC-F* gene (*Camo-F1*), and 18 non-MHC genes, pseudogenes, and non-coding ORFs (Fig 1A and S1 Table).

**Fig 2:**
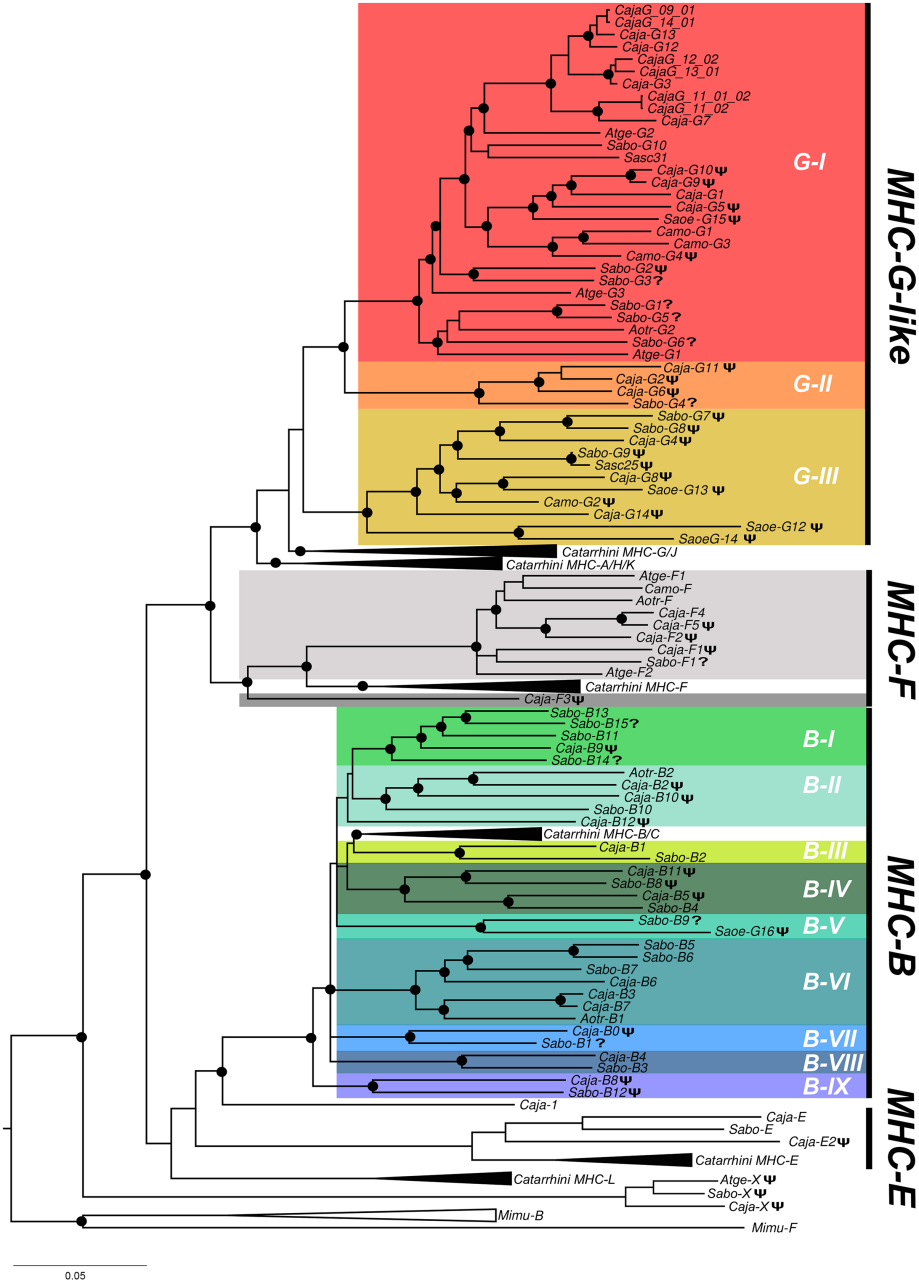
Bayesian phylogenetic tree based on introns 1–8 from primate MHC class I genes and pseudogenes. Black dots at the branching points indicate a support with a posterior probability greater than 0.9. A phi (Ψ) symbol in front of a sequence name indicates pseudogene and a question mark (?) indicates an incompletely sequenced gene. Catarrhini clades include human and Rhesus monkey MHC class I sequences, whereas MHC class I sequences from the grey mouse lemur *Microcebus murinus* (Mimu) were used as outgroup. Lineages are indicated by a G (*MHC-G-like*) or a B (*MHC-B*), followed by a Roman number. Caja, *Callitrhix jacchus*; Sabo, *Saimiri boliviensis*; Sasc, *Saimiri sciureus*; Camo, *Callicebus moloch*; Atge, *Ateles geoffroyi*; Saoe, *Saguinus oedipus*; Aotr, *Aotus trivirgatus*. Accession numbers for published Platyrrhini MHC class I sequences used in the analysis are listed in S3 Table.

In order to have better insights on the diversification dynamics of the Platyrrhini MHC class I region, a Bayesian phylogenetic analysis was performed based on intronic sequences from the genes and pseudogenes presented above. Since introns evolve neutrally, their phylogeny reflects closer the actual evolutionary history of the genes than that based on exons, as they are subjected to natural selection. The phylogenetic tree showed that Platyrrhini *MHC-G-like*, *MHC-F* and *MHC-B* sequences clustered as a sister groups of their Catarrhini counterparts (Fig 2). Platyrrhini *MHC-G-like* sequences formed three well-differentiated clades (G-I, G-II, and G-III), where clade G-I contained the majority of the sequences from the analyzed species, clade G-II included three *C*. *jacchus* pseudogenes and a partial sequence from *S*. *boliviensis* (*Sabo-G4*), and clade G-III included only pseudogenes from the three species analyzed, along with genomic sequences from *C*. *jacchus*, the squirrel monkey (*Saimiri sciureus*), and the cotton top tamarin (*Saguinus oedipus*) found in public databases (Fig 2). Within the clades most sequences cluster with paralogous genes, whereas orthologous *MHC-G-like* genes were hypothesized only for *Sabo-G10/Sasc31*, *Aotr-G2/Sabo-G6/Atge-G1*, *Sabo-G9/Sasc-25*, and *Caja-G8/Saoe-G13*. This suggests that most *MHC-G-like* loci have diversified independently in Platyrrhini taxa by a continuous process of gene duplication and inactivation or deletion, followed by sequence divergence. The pseudogene *MHC-X* in the centromeric end of the alpha block of *C*. *jacchus*, *A*. *geoffroyi*, and *C*. *moloch*, formed a monophyletic group ancestral to all known primate MHC class I loci (Fig 2).

Platyrrhini *MHC-F* genes and pseudogenes were organized as a sister group of Catarrhini sequences, with the exception of the pseudogene *Caja-F3* that was outside of these two groups (Fig 2). The available data suggests that the *MHC-F* locus has duplicated differentially among the Platyrrhini species after the divergence from Catarrhini, with at least one duplication event in *A*. *geofroyii* and various events in *C*. *jacchus*. Platyrrhini *MHC-B* sequences formed nine well-differentiated clades (I-IX), each with at least one sequence from *C*. *jacchus* and *S*. *boliviensis*, except for clade B-V that contained a single sequence from each *S*. *boliviensis* and *S*. *oedipus* (Fig 2). Clades B-III and B-IV were the most closely related to the Catarrhini *MHC-B* loci, although their association in the tree was not statistically well supported. In contrast to *MHC-G-like* sequences, most *MHC-B* sequences clustered with putative orthologous genes, suggesting that most of the duplications that configured the *C*. *jacchus* and *S*. *boliviensis MHC-B* region predated the divergence of these two species, approximately 17 million MYA [10].

### Diversification of transcriptionally active *MHC-G-like* and *MHC-B loci* in Platyrrhini

**Fig 3:**
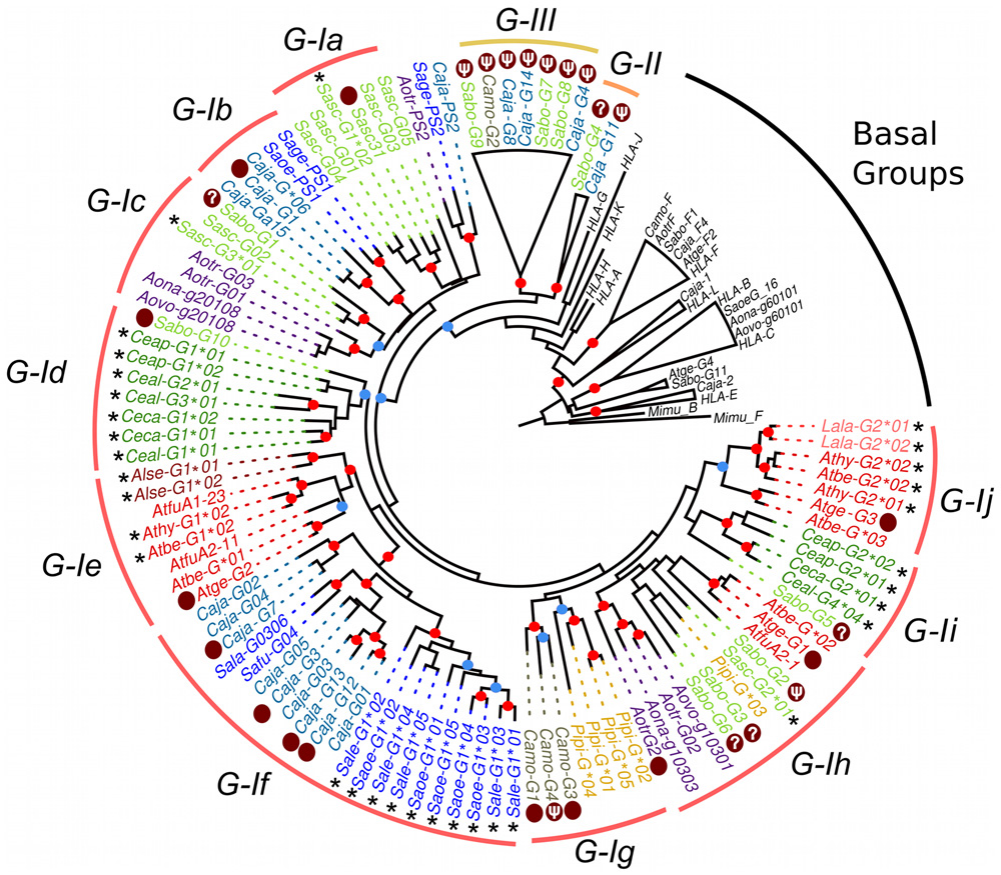
Bayesian phylogenetic tree based on exons 4–8 of Platyrrhini *MHC-G-like* sequences. Sequences marked with an asterisk (*) are cDNAs from this work, whereas those marked with a dark red dot are genomic sequences used to assign cDNAs to a given clade. Red dots on the branching points represent a support with a posterior probability > 0.9, and light blue dots represent a support with a posterior probability between 0.7 and 0.9. Lineages and clusters are indicated at the periphery of the tree. Caja, *Callithrix jacchus*; Sabo, *Saimiri boliviensis*; Sasc, *Saimiri sciureus*; Camo, *Callicebus moloch*; Pipi, *Pithecia pithecia*; Atge, *Ateles geoffroyi*; Atfu, *Ateles fusciceps*; Athy, *Ateles hybridus*; Atbe, *Ateles belzebuth*; Alse, *Alouatta seniculus*; Lala, *Lagothrix lagotricha*; Saoe, *Saguinus oedipus*; Sage, *Saguinus geoffroyi*; Sala, *Saguinus labiatus*; Safu, *Saguinus fuscicollis*; Sale, *Saguinus leucopus*; Aotr, *Aotus trivirgatus*; Aona, *Aotus nancymae*; Aovo, *Aotus vociferans*; Ceap, *Cebus apella*; Ceal, *Cebus albifrons*; Ceca, *Cebus capucinus*. Accession numbers for published Platyrrhini MHC class I sequences used in the analysis are listed in S3 Table.

In order to evaluate the diversity of transcriptionally active MHC class I loci in Platyrrhini, *MHC-G-like* and-*B* cDNAs were cloned and sequenced in a single individual from nine species from the families *Atelidae* (*Ateles belzebuth*, *A*. *hybridus*, *Lagothrix lagotricha*, and *Alouatta seniculus*) and *Cebidae* (*Cebus apella*, *C*. *capucinus*, *Saimiri sciureus*, *Saguinus oedipus* and *S*. *leucopus*). Sequencing 24 plasmid clones per individual resulted in two to five unique cDNAs from each *MHC-G-like* and *MHC-B* locus, with the exception of the two *Saguinus* species (*S*. *leucopus* and *S*. *oedipus*) where no *MHC-B* cDNAs were identified (Table 1; GenBank accession numbers KM219682—KM219731). A phylogenetic analysis of exons 4–8 of these *MHC-G-like* cDNAs, together with sequences obtained from public databases, was carried out in order to have a better representation of the evolutionary history of the genes, given that these exons are not subjected to diversifying selection. The analysis showed that the sequences clustered only within the G-I clade identified in the analysis of genomic sequences (Fig 3). Within clade G-I, the cDNAs grouped in 10 distinctive clusters (a-j), with all clusters containing sequences from a single Platyrrhini family, with the exception of cluster G-Ih that had sequences from the three Platyrrhini families, *Atelidae*, *Cebidae*, and *Pithecidae*. *MHC-G-like* cDNAs from the *Atelidae* family grouped in clusters G-Ih, G-Ie, and G-Ij, with the latter two clusters containing sequences from two *Atelidae* genera. *MHC-G-like* cDNAs from the *Cebidae* family tended to form genus-specific clusters, where sequences from the genus *Cebus* grouped in clusters G-Id and G-Ii, *Saimiri* sequences grouped in clusters G-Ia, -Ic, -Ih, *Aotus* sequences in clusters G-Ic and —Ih, *Callithrix* sequences in clusters G-Ib and —If, and *Saguinus* sequences in cluster If (Fig 3). Finally, *Pitheciidae MHC-G-like* sequences (*Pithecia pithecia and C*. *moloch*) grouped in cluster G-Ig and -Ih. Thus, *MHC-G-like* cDNAs had a general tendency to cluster by families or genera, indicating that this locus has diversified relatively recently after the divergence of such taxa.

**Table 1:** *MHC-G-like* and–*B* cDNAs identified in representative species of the Platyrrhini families *Atelidae* and *Cebidae*.

Family	Subfamily	Species	MHC locus	Allele	Clade
*Atelidae*	*Alouttinae*	*Alouatta seniculus*	*G-like*	Alse-G1*01	G-Ie
			*G-like*	-G1*02	G-Ie
			*B*	-B1*01	B-I
			*B*	-B1*02	B-I
			*B*	-B2*01	B-V
	*Atelinae*	*Ateles belzebuth*	*G-like*	Atbe-G1*02	G-Ie
			*G-like*	-G2*02	G-Ij
			*B*	-B1*02	B-I
			*B*	-B1*03	B-I
			*B*	-B2*01	B-V
		*Ateles hybridus*	*G-like*	Athy-G1*02	G-Ie
			*G-like*	-G2*02	G-Ij
			*G-like*	-G2*01	G-Ij
			*B*	-B1*01	B-I
			*B*	-B1*02	B-I
			*B*	-B2*01	B-V
		*Lagothrix lagotricha*	*G-like*	Lala-G2*01	G-Ij
			*G-like*	-G2*02	G-Ij
			*B*	-B1*01	B-I
			*B*	-B1*02	B-I
			*B*	-B2*01	B-V
			*B*	-B2*02	B-V
*Cebidae*	*Cebinae*	*Cebus apella*	*G-like*	Ceap-G1*01	G-Id
			*G-like*	-G1*02	G-Id
			*G-like*	-G2*01	G-Ii
			*G-like*	-G2*02	G-Ii
			*B*	-B1*01	B-I
			*B*	-B1*02	B-I
		*Cebus capucinus*	*G-like*	Ceca-G1*01	G-Id
			*G-like*	-G1*02	G-Id
			*G-like*	-G2*01	G-Ii
			*B*	-B1*01	B-VI
			*B*	-B1*02	B-I
	*Saimirinae*	*Saimiri sciureus*	*G-like*	Sasc-G1*02	G-Ia
			*G-like*	-G2*01	G-Ih
			*G-like*	-G3*01	G-Ic
			*B*	-B1*01	B-I
			*B*	-B1*02	B-I
			*B*	-B2*01	B-V
			*B*	-B2*02	B-V
	*Callitrichinae*	*Saguinus leucopus*	*G-like*	Sale-G1*01	G-If
			*G-like*	-G1*02	G-If
			*G-like*	-G1*03	G-If
			*G-like*	-G1*04	G-If
			*G-like*	-G1*05	G-If
		*Saguinus oedipus*	*G-like*	Saoe-G1*03	G-If
			*G-like*	-G1*01	G-If
			*G-like*	-G1*02	G-If
			*G-like*	-G1*05	G-If
			*G-like*	-G1*04	G-If

**Fig 4:**
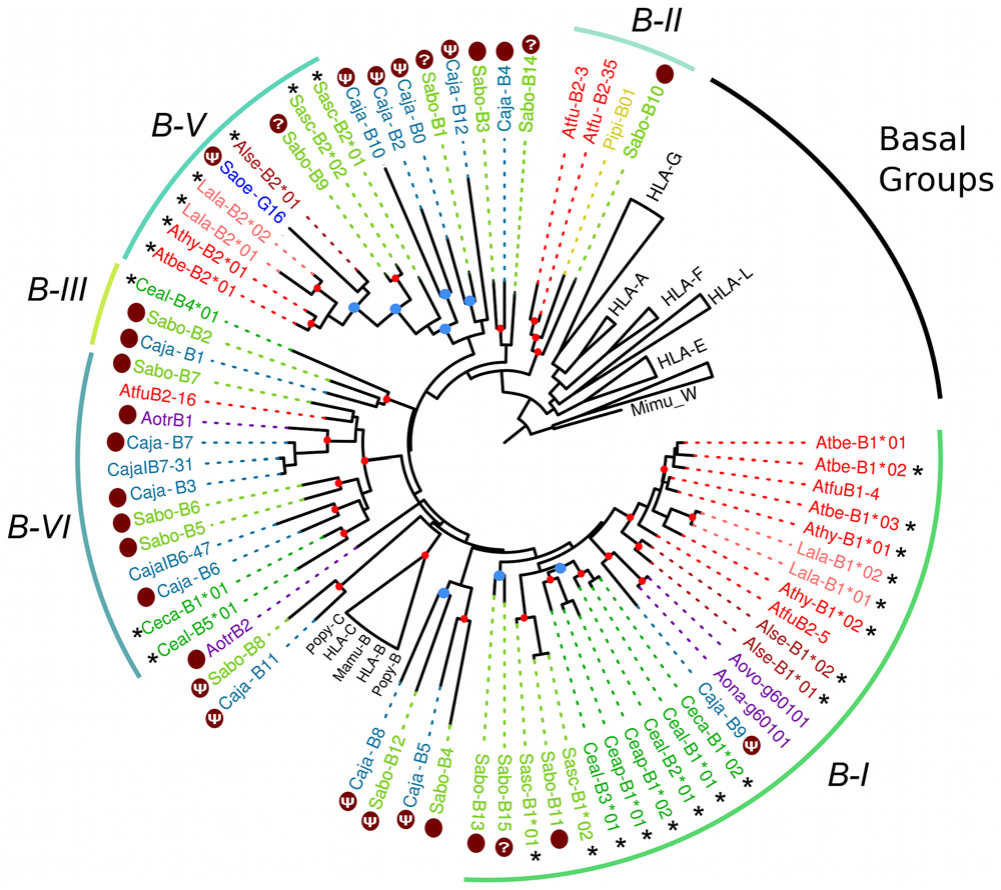
Bayesian phylogenetic tree based on exons 4–8 of Platyrrhini *MHC-B* sequences. Sequences marked with an asterisk (*) are cDNAs from this work, whereas those marked with a dark red dot are genomic sequences used to assign cDNAs to a given lineage. Red dots on the branching points represent a support with a posterior probability > 0.9, and light blue dots represent a support with a posterior probability between 0.7 and 0.9. Lineages and clusters are indicated at the periphery of the tree. Caja, *Callithrix jacchus*; Sabo, *Saimiri boliviensis*; Sasc, *Saimiri sciureus*; Pipi, *Pithecia pithecia*; Athy, *Ateles hybridus*; Atbe, *Ateles belzebuth*; Atfu, *Ateles fusciceps*; Alse, *Alouatta seniculus*; Lala, *Lagothrix lagotricha*; Ceap, *Cebus apella*; Ceal, *Cebus albifrons*; Ceca, *Cebus capucinus*; Aotr, *Aotus trivirgatus*; Aona, *Aotus nancymae*; Aovo, *Aotus vociferans*; Saoe, *Saguinus oedipus*. Accession numbers for published Platyrrhini MHC class I sequences used in the analysis are listed in S3 Table.

A gene tree of primate *MHC-B* sequences showed that the Platyrrhini cDNAs clustered in five clades (I–VI) out the nine defined with the genomic sequences (Fig 4). These clades, with the exception of B-III that had a single cDNA, contained sequences from two different Platyrrhini families, either *Atelidae* and *Cebidae* or *Atelidae* and *Pitheciidae*. Cluster B-I contained 57% of the analyzed cDNAs (21 out of 37) and included sequences from the families *Atelidae* (genera *Ateles*, *Lagothrix*, *Alouatta*) and *Cebidae* (genera *Cebus*, *Saimiri* and *Aotus*). Clade B-II contained sequences from the families *Atelidae* (genus *Ateles*) and *Pitheciidae* (genus *Pithecia*), clade B-III had a single cDNA from the *Cebidae* family (*Cebus*), clade B-V included seven cDNAs from the families *Atelidae* (*Ateles*, *Lagothrix*, *Alouatta*) and *Cebidae* (*Saimiri*), and clade B-VI had cDNA sequences from the *Atelidae* (*Ateles*) and *Cebidae* (*Callithrix* and *Cebus*) families (Fig 4). Hence, the clustering pattern of *MHC-B* and *MHC-G-like* cDNAs differs in that the former shows five major clades that include sequences from different Platyrrhini families, whereas the latter displays only one major clade forming groups composed by cDNAs from a single family or even from a single genus. This indicates that *MHC-G-like* loci have diversified more recently than *MHC-B* loci, and that this rapid diversification has often coincided with the adaptive radiation of the Platyrrhini genera [13].

### Polymorphism of *MHC-G-like* and *MHC-B* loci in the capuchin monkey Cebus albifrons

**Fig 5:**
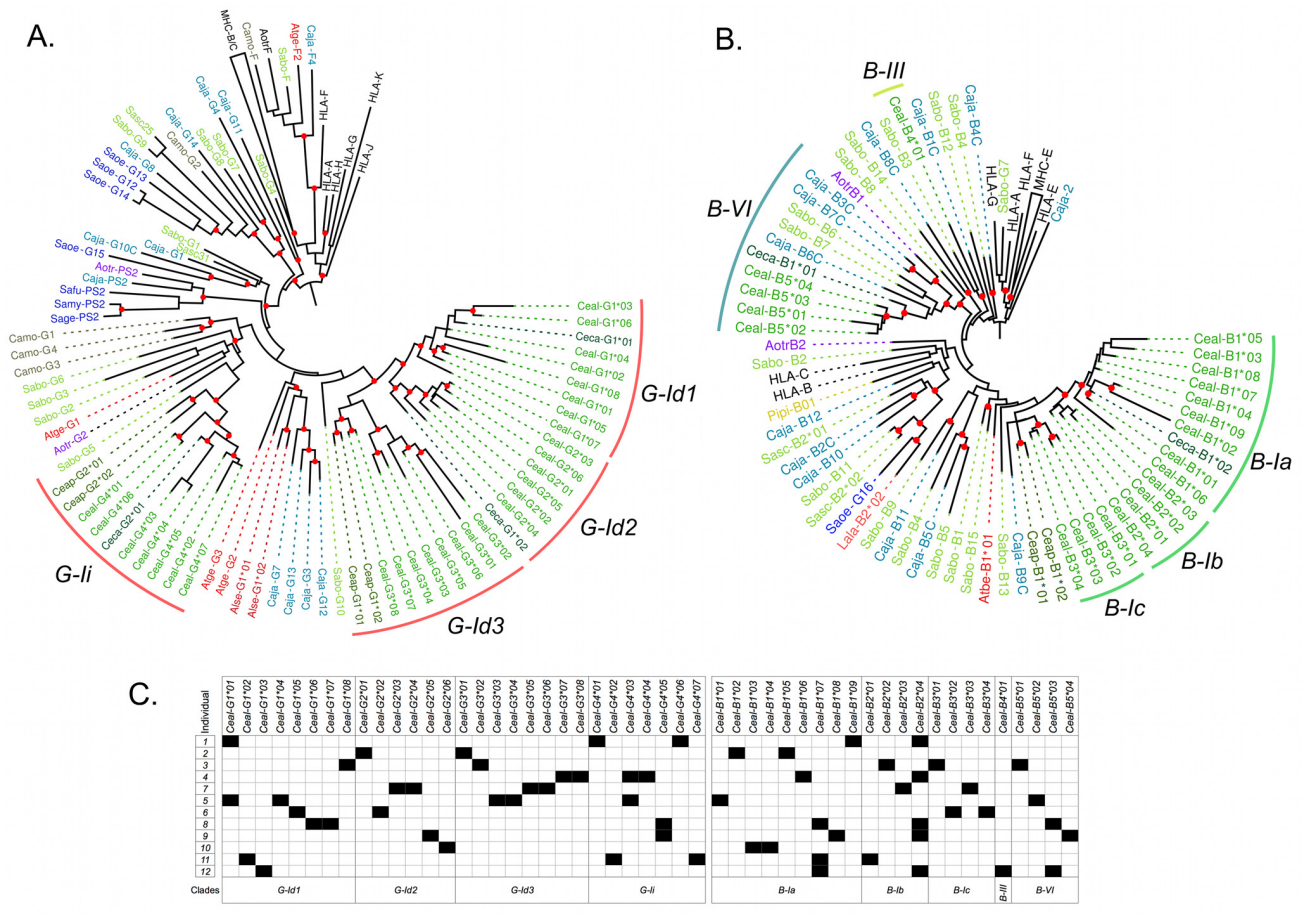
Diversification of *MHC-G-like* and *MHC-B* loci in a population of 12 unrelated white-fronted capuchin monkeys (*Cebus albifrons*). Bayesian phylogenetic trees of *MHC-G-like* (A) and *MHC-B* (B) cDNAs based on exons 4–8, showing at the periphery of the tree the lineages and clusters as defined in the text. Red dots on branching points indicate a support with a posterior probability > 0.7. C, Assignment of putative alleles to each individual of the population, where black squares indicate presence and white squares indicate absence.

MHC class I polymorphism in the capuchin monkey (*C*. *albifrons)* was assessed by cloning and sequencing cDNAs from 12 unrelated individuals. The MHC class I cDNAs derived from PCR amplifications using generic primers designed to amplify both *MHC-G-like* and-*B* loci. The resulting sequences were 1,041 bp long, included exon 1 to 8, and had no alterations that might prevent their proper translation. In total, 29 unique *MHC-G-like* cDNAs were identified in this population, with one to five sequences per individual, and a mean sequence distance of *d* = 9 ± 0.6% (GenBank accession numbers KM219732—KM219782). Twenty-six out of the 29 sequences were not shared between individuals whereas the other three cDNAs where shared by two individuals each (Fig 5). A phylogenetic tree of *C*. *albifrons MHC-G-like* cDNAs showed that they grouped in the two clusters exclusive for the genus Cebus, G-Id and G-Ii, having a mean inter-cluster distance of *d* = 11.5 ± 1% (Fig 5A). The G-Id clade was constituted by three groups with *C*. *albifrons* sequences, G-Id1, G-Id2, and G-Id3, with an average within-group distance of 8.1 ± 0.6%, 5.9 ± 0.6%, and 5.8 ± 0.5%, respectively. Only one group was distinguished in clade G-Ii, having an average within-group distance of *d* = 5.8 ± 0.4% (Fig 5A). These four sequence groups might represent the transcribed *MHC-G-like* loci in this population, which is supported not only by the clustering pattern in the phylogenetic tree, but also by the fact that there is no individual with more than two sequences in each group (Fig 5C). However, no individual had representative sequences in all four groups/loci, suggesting that there exist haplotypic variability in gene number among the individuals of this population.

Twenty-two unique *MHC-B* cDNAs were identified in the *C*. *albifrons* population with two to four sequences per individual and with an average sequence distance of *d* = 10.7 ± 0.7%. The majority of sequences (19) were unique of a single individual, whereas three cDNAs were present in two, three, and five individuals, respectively (Fig 5C). *C*. *albifrons* cDNAs clustered in three of the clades identified in the phylogenetic analysis of genomic sequences, B-I, B-III, and B-VI, having a mean distance between clades B-I and B-III of *d* = 17.0 ± 1.4%, between clades B-I and B-VI of *d* = 18.8 ± 1.4%, and between clades B-III and B-VI of *d* = 20.3 ± 1.7. Clade B-I contained 17 of the 22 cDNAs (77.3%) having a mean sequence distance of *d* = 5.8 ± 0.5%. Sequences from clade B-I were in turn organized into three groups, B-Ia, B-Ib, and B-Ic (Fig 5B), having an average within-group distance of 3.7 ± 0.3%, 5.1 ± 0.5%, and 4.8 ± 0.5%, respectively. Clade B-III contained a single cDNA (Ceal-B4*01) and clade B-VI contained four cDNAs clustered together and having an average distance of 4.1 ± 0.5%. As discussed for *MHC-G-like* sequences, it is possible that the five groups segregating in the phylogenetic tree represent different loci, as there are no individuals with more than two cDNAs per group. Also, there are no individuals with *MHC-B* cDNAs in each of the five groups, suggesting that there are different haplotypes in the population varying in gene content.

### Natural Selection has promoted the diversification of MHC-G-like and -B loci in *C*. *albifrons*

**Fig 6:**
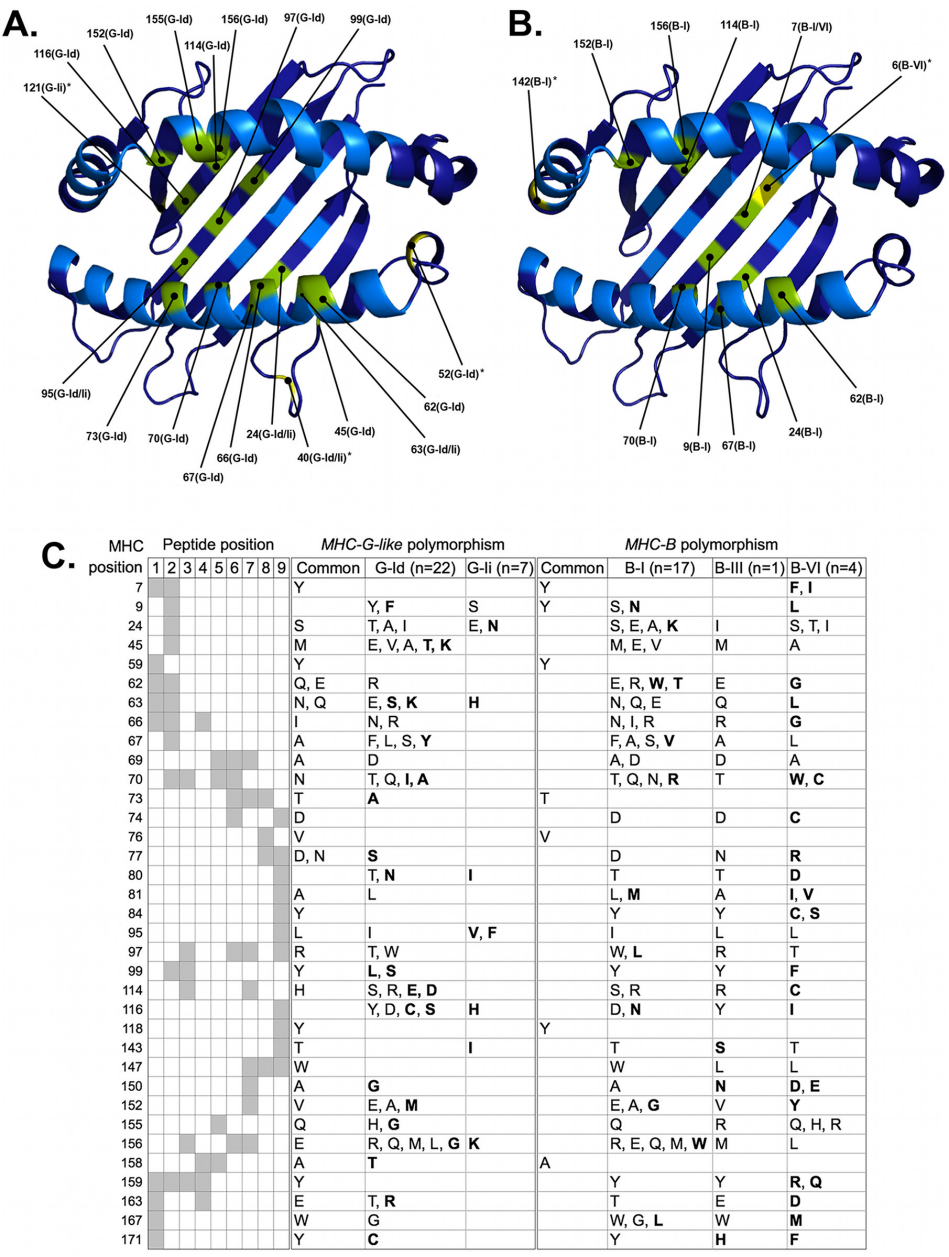
Natural selection has promoted the diversification of *C*. *albifrons* MHC class I loci at PBR positions predicted to interact with the processed peptide. PBR residues evolving under positive selection in Platyrrhini MHC-G-like (A) and MHC-B (B) lineages are indicated on the structural model with their corresponding positions. In parenthesis next to the position number is indicated the particular lineage where positive selection was detected. Residues colored in green are positively selected positions predicted to contact the peptide, whereas residues colored in yellow and marked with an asterisk are positively selected positions that do not contact the peptide. C, variability of MHC-G-like and MHC-B positions that interact with the processed peptide within 4.0 Å. In the left-hand panel, the rows give the MHC numbering positions, the columns give the peptide positions, and the intersections between them (in gray background) mark the predicted interactions between MHC positions and each of the 9 residues of the peptide, according to [35]. The center and right-hand panels show the different amino acids at each position of MHC-G-like and MHC-B predicted proteins, respectively. Residues shared by lineages and those unique for a lineage within either MHC-G-like or —B are indicated separately. Residues in bold type are unique for a lineage and are not shared between MHC-G-like and MHC-B loci.

Natural selection analysis of *MHC-G-like* cDNAs from *C*. *albifrons* resulted in the identification of 19 positively selected positions from the peptide binding region (PBR), 16 of which have been predicted to interact with the peptide within 4.0 Å [35] (Fig 6). Three of the positively selected positions contacting the peptide were identified in both G-Id and G-Ii lineages (positions 24, 63, and 95), whereas 13 of such positions (45, 62, 66, 67, 70, 73, 97, 99, 114, 116, 152, 155, and 156) were unique of the G-Id lineage but none for of the G-Ii lineage (Fig 6A). *MHC-G-like* cDNAs encoded two or more alternative amino acids in 27 of the 35 peptide-contacting positions, although the majority of variable positions occurred in the G-Id lineage (26) in comparison to the G-Ii lineage (7) (Fig 6C). In turn, MHC-B predicted proteins had 11 positively selected residues in the PBR and nine of them contacting the peptide within 4.0 Å (Fig 6B). One of the latter positions (position 7) was shared by lineages B-I and B-VI, eight positions (9, 24, 62, 67, 70, 114, 152, and 156) were detected only in lineage B-I, and none was unique for the B-VI lineage. Furthermore, seven positively selected positions contacting the peptide (24, 62, 67, 70, 114, 152, 156) were shared between MHC-G-like and MHC-B predicted proteins (Fig 6A and 6B). *MHC*-B cDNAs encoded 30 variable peptide-contacting positions, 16 from lineage B-I and nine from lineage B-VI (Fig 6C). These MHC-B variable residues tended to be unique of a given clade, in contrast to MHC-G-like predicted proteins where clades shared most of the amino acids in variable positions. Indeed, in 24 out of the 27 MHC-G-like variable peptide-contacting positions, clades G-Id and G-Ii had at least one amino acid in common, whereas in only three of the 30 MHC-B variable positions the three clades had at least one amino acid in common (Fig 6C). However, the number of variable positions sharing amino acids was different between clades, as there were 17 positions with shared amino acids between clades B-I and B-III, six between B-I and B-VI, and six between B-III and B-VI. This suggests that the degree overlapping between peptides presented by MHC-G-like is greater than that of MHC-B molecules, and that the overlapping of peptides presented by proteins of the B-I and B-III clades is greater than that of proteins from clade B-VII with any of the other two MHC-B clades. The most variable peptide-contacting position for both MHC-G-like and —B was position 156, located at the PBR alpha-2 helix and contacting peptide positions 3, 6, and 7, with seven different amino acids for each locus class (Fig 6C). Yet, most other variable positions (e.g., positions 24, 45, 63, 81, 116) were predicted to interact with the peptide anchor positions 2 and 9, suggesting that both MHC-G-like and —B can present a wide spectrum of processed peptides (Fig 6C).

Besides the interaction with peptides and T-cell receptors (TcRs), the PBR of MHC class I molecules also interact with KIRs to modulate the activation of NK cells and some T lymphocyte populations. The KIR Ig domains dock onto the C-terminal end of the PBR recognizing polymorphic regions that determine the specificity of the MHC:KIR binding interaction [29]. Mutational analyses on a HLA-B molecule have shown that substitutions in positions 80, 83,142, 146 and 149 markedly affect the affinity to KIR3DL1 [29]. In *C*. *albifrons* MHC-G-like and —B predicted proteins, 12 of the 14 PBR positions that interact with the KIR D1 and D2 domains were variable, either within or between clades (S2 Table), including the five critical positions identified in the mutational studies. Some of the variants in KIR-interacting positions defined clades, for example, the variants Tyr84, Cys84, and Ser84 differentiated the MHC-B lineages B-I, B-III, and B-VI, respectively, Ile80 was unique of clade G-Id, and Asp80 and Lys151 were unique of clade B-VI (S2 Table). These data suggests that *C*. *albifrons* MHC class I molecules have distinct specificities for KIR molecules.

**Fig 7:**
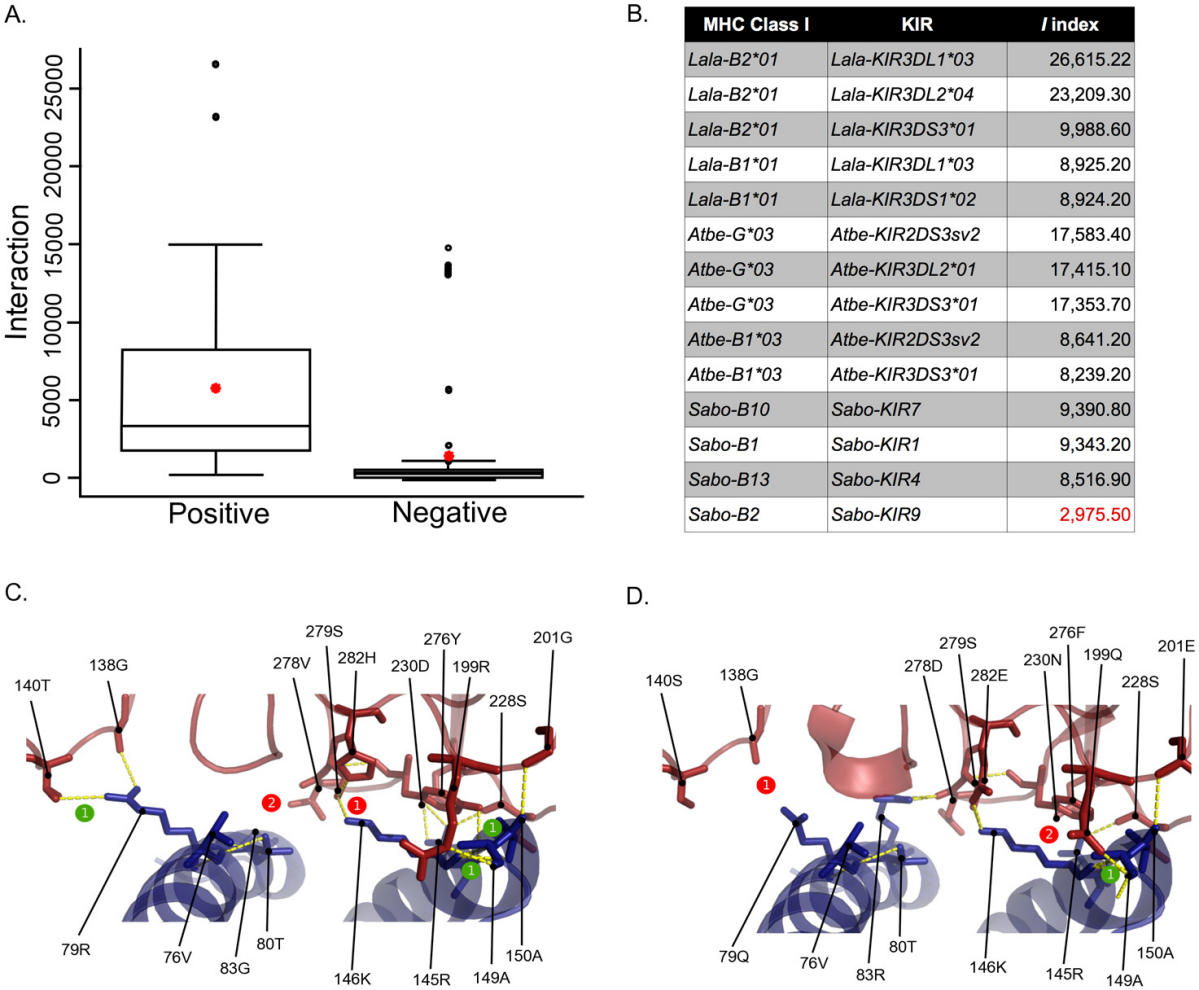
Interaction (*I*) index proposed to predict stable interaction between MHC class I and KIR molecules, based on the number of hydrogen bonds and the desolvation energy. A, Box plot of *I* index values from experimentally validated human, chimpanzee and Rhesus monkey MHC:KIR pairs with functional interactions (positive, n = 45) and lack of interaction (negative, n = 71). The red star indicates the average *I* index value for positive and negative interactions. B, *I* index values derived from models of Platyrrhini MHC:KIR interacting pairs. Structural model of interacting pairs Lala-B2*01:Lala-KIR3DL1*03 with *I* = 26,615 (C) and Sabo-B2:Sabo-KIR9 *I* = 2,975 (D). Number of hydrogen bond gained (numbers in green circles) and lost (numbers in red circles) with respect to the human HLA-B*5701:KIR3DL1 (PDB: 3VH8) interaction are indicated along with the MHC class I and KIR residues critical for the interaction [29]. Accession numbers for the Platyrrhini KIR sequences used in this analysis are in the S3 Table.

In order to identify potential MHC:KIR interacting pairs in Platyrrhini, an interaction index *I = kh / ln dG* was proposed. This index takes into account the average energy of an H-bond (k), the number of H-bonds in the pair (h), and the desolvation energy (dG). The index was first applied to 116 human, chimpanzee and Rhesus monkey MHC:KIR pairs that had been experimentally evaluated. The mean *I*-value for interacting pairs was 5,250 (n = 45) and that for non-interacting pairs was 1,376 (n = 71) (Fig 7A). Some positive interactions had low *I*-values and some negative interactions had high *I*-values (i.e., false positives and false negatives), representing 15% of the 116 interactions. Then, MHC:KIR pairs were evaluated for Platyrrhini species with available sequence information for both molecules [36], revealing some potential interacting pairs, including cases of an MHC class I molecule interacting with more than one KIR (Fig 7B). Structural modeling of MHC:KIR pairs with a high and a low *I* index (Lala-B2*01:Lala-KIR3DL1*03 and Sabo-B2:Sabo-KIR9), revealed a much more stable interaction in the *L*. *lagotricha* pair having a *I* = 26,615 (Fig 7C) than in its *S*. *boliviensis* counterpart with a *I* = 2,975 (Fig 7D). This indicates that the interaction index proposed here has the power to predict MHC:KIR interactions based on protein sequences.

## Discussion

For many years, the study of the MHC class I system in Platyrrhini species focused mostly on characterizing the diversity of *MHC-G-like* loci. Although few studies indicated the presence of *MHC-B* loci in Platyrrhini, the impressive complexity of the region in this taxon was only realized until the sequencing of the MHC class I beta block in the common marmoset (*C*. *jacchus*) [16]. Moreover, unexpected complexity was also found in the common marmoset MHC class I alpha block, with more than a dozen *MHC-G-like* and five *MHC-F* genes and pseudogenes [15]. This great expansion of the *MHC-G-like* and *MHC-B* loci is here reported for another Cebidae species, the Bolivian squirrel monkey (*S*. *boliviensis*), which contained at least 10 *MHC-G-like* sequences (five putative genes and five pseudogenes) and 15 *MHC-B* sequences (13 putative genes and two pseudogenes). This high number of MHC class I sequences identified in the squirrel monkey’s draft genome is unlikely to be an assembly artifact. Assembly of high depth Illumina-based sequenced genomes typically results in underrepresentation of recent, rapidly evolving, lineage-specific genomic regions, due to the collapse of segmental duplications and recent repeat regions [37]. Therefore, assembly artifacts would involve missing segmental duplications instead of increasing the number of duplicates. The expansion of the MHC class I subgenomic regions, however, does not appear to be a common trait to all Platyrrhini species. Indeed, the *MHC-G-like* region of the spider monkey (*A*. *geoffroyi*), which was completely covered by two BAC clones, contained only three *MHC-G-like* and two *MHC-F* genes. Differential expansion of the MHC class I genes between related species has been previously observed. Humans and apes contain single *HLA-A* and–*B* genes whereas Rhesus monkeys contain multiple copies of both *MHC-A* and *MHC-B* loci [9]. Similarly, in Galliform birds, the gene number and organization of MHC class I genes varies between closely related species [38]. Thus, the MHC class I alpha and beta blocks in Platyrrhini have evolved under a birth-and-death model where some taxa have undergone a rapid process of gene or regional duplications, whereas others have maintained a low copy number either as an ancestral condition or as a consequence of a taxon-specific gene deletions. The process of regional expansion evidenced in *Cebidae* species appears to be more accelerated in the alpha block than in the beta block as can be inferred by the limited number of orthologous *MHC-G-like* genes in comparison with the more frequent instances of orthology between *MHC-B* loci. The high rate of diversification of the MHC class I alpha and beta blocks contrasts with the highly conserved kappa block containing the *MHC-E* locus, which has remained virtually unchanged since the divergence of Catarrhini and Platyrrhini (S1 Fig).

In most of the species analyzed in this work there was evidence for various transcriptionally active *MHC-G-like* and–*B* loci, having a number of unique cDNAs per individual that raged from two to five for both classes of loci. In the two *Callitrichinae* species analyzed (*S*. *oedipus* and *S*. *leucopus*), however, no *MHC-B* cDNAs were found, although five *MHC-G-like* cDNAs were identified in each species. A similar case has been reported in the common marmoset, also a Callitrichinae, that, despite having several *MHC-B* genes, no evidence for *MHC-B* transcripts was found [17]. Yet, with more sequencing effort *MHC-B* has been detected in this species [16]. Hence, it is possible that the *MHC-B* loci in *Callitrichinae* species have limited expression levels in peripheral blood cells and/or their expression is not universal but restricted to selected tissues.

The number of MHC class I cDNAs per species identified in this work may appear low in light of the large number of genes exhibited by some Platyrrhini species. Although *S*. *boliviensis* and *A*. *geoffroyi* were not sampled, their sister species (*S*. *sciureus* and *A*. *belzebuth* / *A*. *hybridus*) showed two or three *MHC-G-like* cDNAs and three or four *MHC-B* cDNAs. Assuming that the complexity of the MHC class I region of *S*. *boliviensis* and *A*. *geoffroyi* is similar to that of their sister species, it can be hypothesized that the number of MHC class I loci with high transcriptional activity in Platyrrhini species is small and relatively fixed (two to four), regardless of the MHC class I genomic complexity of the species. This pattern has been observed in the highly expanded MHC class I region of the Rhesus monkey, where there are few *MHC-A* and–*B* genes with high expression levels with the remaining genes displaying low expression levels [39]. However, it is also possible that the generic primers used to amplify cDNAs were not efficient enough to amplify the entire set of *MHC-G-like* and–*B* sequences from the sampled species. Solving this issue would require access to genomic sequences to design locus-specific primers and a quantitative methodology to evaluate differential expression of genes.

The analysis of MHC class I diversity in 12 unrelated *C*. *albifrons* individuals showed a maximum five *MHC-G-like* and four *MHC-B* cDNAs per individual. The vast majority of cDNAs were unique for a given individual, although there were sequences shared by up to five individuals, indicating that most MHC class I loci are highly polymorphic. In gene trees, these cDNAs clustered into four *MHC-G-like* and five *MHC-B* groups, likely corresponding to the highly transcribed loci. Not all individuals had representative cDNAs in each group, suggesting that there is haplotypic diversity in both families MHC class I genes. Haplotypic diversity of MHC class I genes has been described in several species, including the Rhesus monkey [39], cattle [40], seals [41], and the zebra fish [42]. The variability in the MHC class I region in Platyrrhini has likely been promoted by natural selection for ligand binding. In *C*. *albifrons*, positions of the PBR predicted to interact with the processed peptide and the Ig domains of KIR molecules were generally highly variable and with signatures of positive selection within the major sequence clades. This indicates that most of the *C*. *albifrons MHC-G-like* and–*B* loci act as classical loci presenting peptides to TcRs and modulating the activity of NK cells.

The MHC class I system in Platyrrhini has been traditionally seen as a product of a relatively rapid diversification of an ancestral locus related to *MHC-G* or *MHC-A*, which occurred after the divergence of Platyrrhini and Catarrhini. The genomic analysis of the common marmoset MHC class I region [15] and the present work unveiled an unsuspected complexity of this gene family in Platyrrhini characterized by a differential expansion and contraction of *MHC-G-like* and *MHC-B* genes between species. This process, that appears to be more rapid in the *MHC-G-like* region than in the *MHC-B* region, generates loci with different transcriptional activities, which might be correlated with different functional roles. Thus, the variability of the MHC class I system is generated at different levels, from genetic polymorphism to gene number variation and differential expression of loci.

## Supporting Information

S1 Fig Draft genomic map of the MHC class I kappa block (*MHC-E*) of *Saimiri boliviensis* compared to its syntenic region in humans and the common marmoset. (TIF)

S1 Table Annotation of draft BAC and scaffold sequences from the alpha block (*MHC-A/G/F*) of *Ateles geoffroyi*, *Callicebus moloch*, and *Saimiri boliviensis* and from the beta block (*MHC-B/C*) of *Saimiri boliviensis*. (DOCX)

S2 Table Variability of MHC-G-like and —B positions predicted to interact with KIR domains D1 and D2 in *C*. *albifrons*. (DOCX)

S3 Table Additional Platyrrhini MHC class I sequences used for phylogenetics analyses. (DOCX)
