# Supplementary figures and images for: Patterns of MHC-G-Like and MHC-B Diversification in New World Monkeys
Source: PLoS One. 2015 Jun 29;10(6):e0131343. doi: 10.1371/journal.pone.0131343 (PMC4486459; doi:10.1371/journal.pone.0131343)

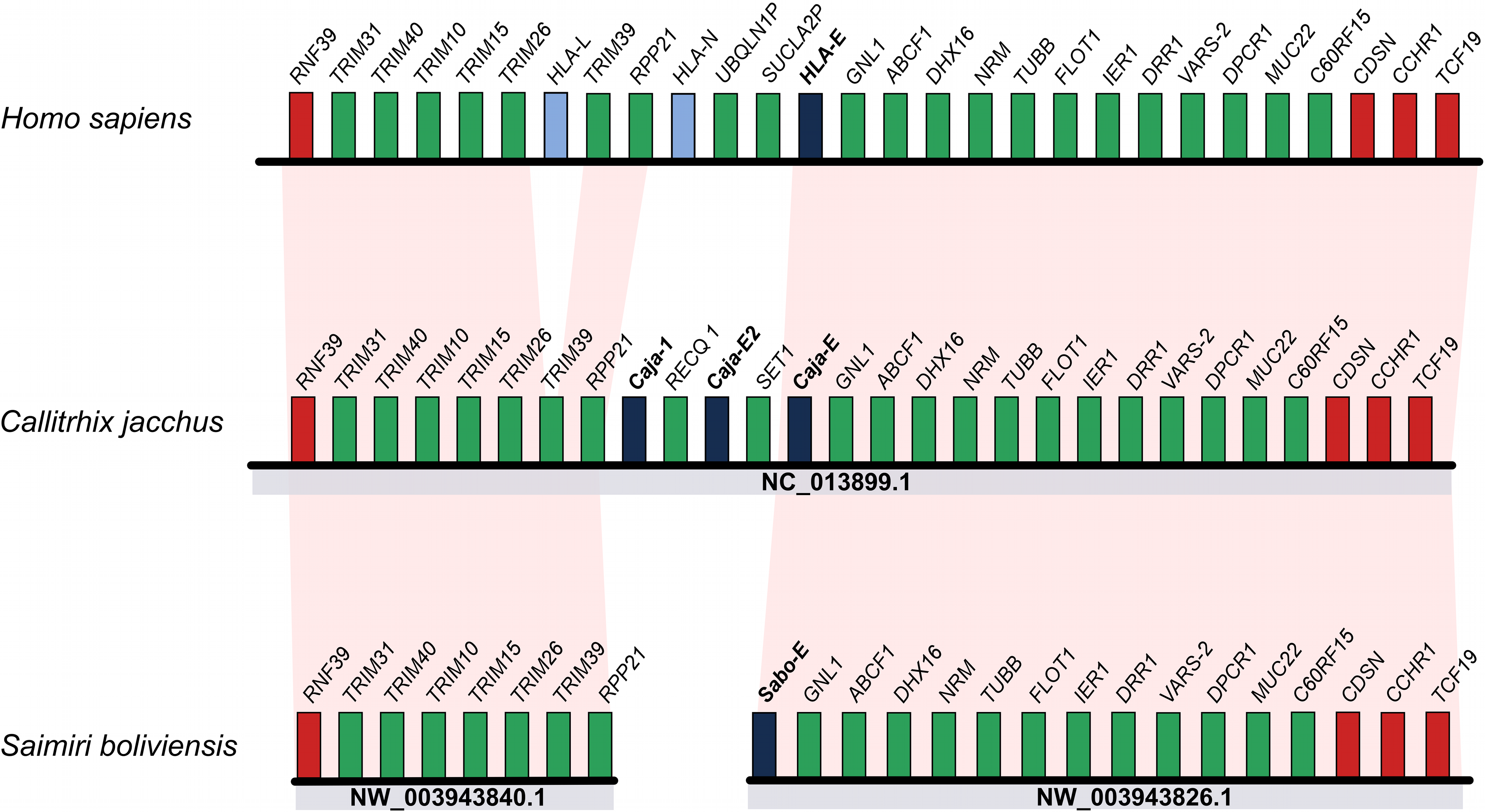

Supplement: S1 Fig — (TIF) [file pone.0131343.s001.tif]
