# Supplementary material for: Patterns of MHC-G-Like and MHC-B Diversification in New World Monkeys
Source: PLoS One. 2015 Jun 29;10(6):e0131343. doi: 10.1371/journal.pone.0131343 (PMC4486459; doi:10.1371/journal.pone.0131343)
Supplement: S1 Table — (DOCX) [file pone.0131343.s002.docx]

**S1 Table.** Annotation of draft BAC and scaffold sequences from the MHC class I alpha block (*MHC-A/G/F*) of *Ateles geoffroyi, Callicebus moloch*, and *Saimiri boliviensis* and the beta block (*MHC-B/C*) of *Saimiri boliviensis.*

| Species | BAC/Scaffold ID | Annotation | Begins^a^ | Ends |
| --- | --- | --- | --- | --- |
| *Saimiri boliviensis* | NW_003943887.1 | *Myelin oligodendrocyte glycoprotein (MOG)* | 91992 | 109577 |
|  |  | *Zinc Finger Protein (ZNF57)* | 83104 | 91537 |
|  |  | *NonHistone Chromosome Protein (NHP2)* | 54507 | 55060 |
|  |  | *Mitochondrial coiled-coil domain 1 (MCCD1)* | 40094 | 40162 |
|  |  | *Sabo-G1* | 5148 | 7373 |
|  |  | *HLA complex group 8 (HCG8), non-coding RNA* | 1 | 1716 |
|  | NW_003943835.1 | *Sabo-G2* | 481060 | 483634 |
|  |  | *Dihydrofolate reductase-like 1 (DHFRL1)* | 474259 | 477719 |
|  |  | *NHP2-like protein 1-like* | 467615 | 461488 |
|  |  | *Mitochondrial coiled-coil domain 1 (MCCD1)* | 448360 | 448802 |
|  |  | *ZNRD1 antisense RNA 1 (ZNRD1-AS1)* | 432012 | 433646 |
|  |  | *Sabo-G3* | 418705 | 420608 |
|  | NW_003943876.1 | *ZNRD1 antisense RNA 1 (ZNRD1-AS1)* | 115293 | 117010 |
|  |  | *HLA complex P5 (HCP5)* | 114652 | 117016 |
|  |  | *Heat shock Protein 90kDa (HSP90)* | 110322 | 109208 |
|  |  | *Heat shock Protein 90kDa (HSP90)* | 109144 | 108045 |
|  |  | *SH3-domain kinase binding protein 1 (SH3KBP1)* | 107913 | 107043 |
|  |  | *Sabo-G4* | 98741 | 99970 |
|  |  | *Dihydrofolate reductase-like 1 (DHFRL1)* | 94154 | 92500 |
|  |  | *HLA complex group 8 (HCG8), non-coding RNA* | 72225 | 73071 |
|  |  | *HLA complex group 4 (HCG4), non-coding RNA* | 63086 | 64423 |
|  |  | *Sabo-F* | 62046 | 63818 |
|  |  | *HLA complex group 4 (HCG4), non-coding RNA* | 5911 | 6673 |
|  |  | *HLA complex group 8 (HCG8), non-coding RNA* | 188 | 890 |
|  | NW_003943863.1 | *HLA complex group 4 (HCG4), non-coding RNA* | 22665 | 23948 |
|  |  | *Sabo-G5* | 24828 | 26698 |
|  |  | *HLA complex group 8 (HCG8), non-coding RNA* | 30421 | 31863 |
|  |  | *Dihydrofolate reductase-like 1 (DHFRL1)* | 37152 | 38553 |
|  |  | *Nucleophosmin (NPM1)* | 61823 | 63548 |
|  |  | *Heterogeneus nuclear riboprotein A1 (HNRNPA1)* | 99400 | 105371 |
|  |  | *HLA complex group 4 (HCG4), non-coding RNA* | 130499 | 131521 |
|  |  | *Sabo-G6* | 132368 | 134769 |
|  |  | *HLA complex group 8 (HCG8), non-coding RNA* | 138130 | 139669 |
|  |  | *MCCD1* | 165175 | 166346 |
|  | NW_003943949.1 | *Sabo-G7* | 14916 | 16996 |
|  | NW_003943840.1 | *SH3-domain kinase binding protein 1 (SH3KBP1)* | 4102 | 7410 |
|  |  | *HLA complex P5 (HCP5)* | 6113 | 7872 |
|  |  | *DNA ligase* | 8398 | 9705 |
|  |  | *HLA complex group 4 (HCG4), non-coding RNA* | 12919 | 13862 |
|  |  | *Sabo-G8* | 14411 | 16830 |
|  |  | *HLA complex group 8 (HCG8), non-coding RNA* | 20051 | 21208 |
|  |  | *HLA complex group 8 (HCG8), non-coding RNA* | 37379 | 38354 |
|  |  | *Heat shock protein 90kDa (HSP90)* | 55991 | 63176 |
|  |  | *SH3-domain kinase binding protein 1 (SH3KBP1)* | 74372 | 76199 |
|  |  | *Sabo-G9* | 82148 | 83691 |
|  |  | *HLA complex group 8 (HCG8), non-coding RNA* | 86477 | 88232 |
|  |  | *HLA complex group 4 (HCG4), non-coding RNA* | 122510 | 124630 |
|  |  | *Sabo-G10* | 123801 | 126560 |
|  |  | *HLA complex group 8 (HCG8), non-coding RNA* | 129325 | 130265 |
|  |  | *Eukaryotic peptide chain release factor 1 (ETFP1)* | 157562 | 159574 |
|  |  | *RPA12* | 159061 | 159952 |
|  |  | *PPR1P11* | 164337 | 167345 |
|  |  | *Sabo-X* | 170338 | 172750 |
|  |  | *RNF39* | 170720 | 182876 |
|  |  | *Ribosomal protein L7-like 1 (RPL7L1)* | 173684 | 174730 |
|  |  | *Putative uncharacterized protein ZNRD1-AS1-like* | 185109 | 186772 |
|  |  |  |  |  |
|  | NW_003943835.1 | *Sabo-B15* | 1 | 2137 |
|  |  | *Sabo-B14* | 35244 | 35836 |
|  |  | *Sabo-B14* | 36830 | 37632 |
|  |  | *Methionine adenosyl transferase II (MAT2A)* | 80000 | 80697 |
|  |  | *Sabo-B13* | 94513 | 97222 |
|  |  | *Sabo-B12* | 149675 | 153747 |
|  |  | *Sabo-B11* | 164117 | 166232 |
|  |  | *Sabo-B11* | 166412 | 167101 |
|  |  | *Cyclin E1 (CCNE1), mRNA* | 226863 | 228236 |
|  |  | *Cyclin E1 (CCNE1), mRNA* | 242438 | 242585 |
|  |  | *Cyclin E1 (CCNE1), mRNA* | 242937 | 243938 |
|  |  | *Sabo-B10* | 273836 | 276781 |
|  |  | *Sabo-B9* | 333153 | 334634 |
|  |  | *Nuclear cap-binding protein subunit 2-like (NCBP2)* | 356949 | 357417 |
|  |  | *Dihydrofolate reductase-like 1 (DHFRL1)* | 414183 | 416510 |
|  |  | *Sabo-G3* | 418705 | 420608 |
|  |  | *ZNRD1 antisense RNA 1 (ZNRD1-AS1)* | 432012 | 433646 |
|  |  | *Mitochondrial coiled-coil domain 1 (MCCD1), mRNA* | 448360 | 448802 |
|  |  | *NHP2-like protein 1-like* | 467615 | 461488 |
|  |  | *Dihydrofolate reductase-like 1 (DHFRL1)* | 474259 | 477719 |
|  |  | *Sabo-G2* | 481060 | 483634 |
|  |  | *Mitochondrial coiled-coil domain 1 (MCCD1), mRNA* | 524559 | 524870 |
|  |  | *Cyclin E1 (CCNE1), mRNA* | 530567 | 531845 |
|  |  | *Cyclin E1 (CCNE1), mRNA* | 532173 | 532488 |
|  |  | *Cyclin E1 (CCNE1), mRNA* | 546044 | 547128 |
|  |  | *Ring finger protein 34* | 559484 | 560472 |
|  |  | *Sabo-B7* | 573079 | 575952 |
|  | NW_003943627.1 | *Sabo-B6* | 86418 | 83557 |
|  |  | *Ring finger protein 34 (RNF34)* | 177628 | 176001 |
|  |  | *Sabo-B5* | 202353 | 199656 |
|  |  | *Sabo-B4* | 234643 | 231900 |
|  |  | *Sabo-B3* | 263160 | 260291 |
|  |  | *Ring finger protein 181 (RNF181), mRNA* | 329636 | 329064 |
|  |  | *Cyclin-E1-like* | 337016 | 334016 |
|  |  | *Cyclin-E1-like* | 347763 | 343763 |
|  |  | *Cyclin-G1-like* | 357743 | 354605 |
|  |  | *Sabo-B2* | 368443 | 364425 |
|  |  | *Ferritin heavy chain-like* | 376020 | 369018 |
|  |  | *Sabo-B1* | 406325 | 404501 |
|  |  | *Keratin, type II cytoskeletal 8-like* | 434443 | 429671 |
|  |  | *MHC class I polypeptide-related sequence A-like* | 448116 | 444838 |
|  |  | *Peptidyl-prolyl cis-trans isomerase A-like* | 478818 | 475390 |
| *Ateles geoffroyi* | AC240700.2 | *Zinc Finger Protein (ZNF57)* | 1829 | 3336 |
|  |  | *Glycine tRNA ligase* | 35538 | 37141 |
|  |  | *Glyciyl tRNA synthetase* | 37143 | 42895 |
|  |  | *Mitochondrial coiled-coil domain 1 (MCCD1)* | 43391 | 44588 |
|  |  | *HLA complex group 26 (HCG26), non-coding RNA* | 46951 | 48090 |
|  |  | ***ZNRD1 antisense RNA 1 (ZNRD1-AS1), non-coding RNA*** | **52365** | **54065** |
|  |  | ***HLA complex group 4 (HCG4), non-coding RNA*** | **62256** | **63965** |
|  |  | ***Atge-G1*** | **63498** | **66216** |
|  |  | ***HLA complex group 8 (HCG8), non-coding RNA*** | **68937** | **70695** |
|  |  | ***Dihydrofolate reductase-like 1 (DHFRL1)*** | **74383** | **76859** |
|  |  | ***Ribosomal protein L26 (RPL26), mRNA*** | **77070** | **77564** |
|  |  | ***Tripartite motif containing 61 (TRIM61), transcript variant X3*** | **80000** | **82436** |
|  |  | ***HLA complex group 4 (HCG4), non-coding RNA*** | **94115** | **96128** |
|  |  | ***Atge-F1*** | **95337** | **98634** |
|  |  | ***HLA complex group 8 (HCG8), non-coding RNA*** | **102327** | **103204** |
|  |  | ***HLA complex group 4 (HCG4), non-coding RNA*** | **110022** | **112038** |
|  |  | ***Atge-F2*** | **111235** | **114485** |
|  |  | ***L1 ERVL*** | **111765** | **114275** |
|  |  | ***HLA complex group 8 (HCG8), non-coding RNA*** | **118294** | **119282** |
|  |  | ***HLA complex group 4 (HCG4), non-coding RNA*** | **155123** | **157137** |
|  |  | ***Atge-G2*** | **156351** | **159116** |
|  |  | ***HLA complex group 8 (HCG8), non-coding RNA*** | **162165** | **163269** |
|  |  | ***HLA complex group 4 (HCG4), non-coding RNA*** | **193401** | **195495** |
|  |  | ***Atge-G3*** | **194678** | **197467** |
|  |  | ***HLA complex group 8 (HCG8), non-coding RNA*** | **200185** | **201161** |
|  | AC241574.2 | *Eukaryotic peptide chain release factor subunit 1 (ETFP1)* | 235811 | 236260 |
|  |  | *Atge-X* | 246423 | 249115 |
|  |  | *Ribosomal Protein L7 Like 1 (RPL7L1)* | 249457 | 250239 |
|  |  | *Putative uncharacterized protein ZNRD1-AS1-like* | 262178 | 265717 |
|  |  | *Protein phosphatase 1 regulatory subunit 11 isoform 1* | 272233 | 277841 |
|  |  | *Ring Finger protein 39 (RNF39)* | 277932 | 278586 |
| *Callicebus moloch* | AC240643.3 | *Myelin oligodendrocyte glycoprotein (MOG)* | 1 | 900 |
|  |  | ***Zinc Finger Protein (ZNF57)*** | **1219** | **6236** |
|  |  | ***HLA complex P5 (HCP5), non-coding RNA*** | **55751** | **58074** |
|  |  | ***HLA complex group 4 (HCG4), non-coding RNA*** | **64077** | **66088** |
|  |  | ***Camo-F*** | **65278** | **67946** |
|  |  | ***HLA complex group 8 (HCG8), non-coding RNA*** | **72339** | **73822** |
|  |  | ***HERVK*** | **82996** | **108575** |
|  |  | ***MCCD1*** | **125157** | **125704** |
|  |  | ***HLA complex group 26 (HCG26), non-coding RNA*** | **128711** | **129854** |
|  |  | ***HLA complex group 4 (HCG4), non-coding RNA*** | **159268** | **161306** |
|  | AC240538.3 | ***Camo-G1*** | **160475** | **163228** |
|  |  | *HLA complex group 8 (HCG8), non-coding RNA* | 166334 | 167308 |
|  |  | *SH3-domain kinase binding protein 1 (SH3KBP1)* | 172406 | 173286 |
|  |  | *HLA complex group 4 (HCG4), non-coding RNA* | 176960 | 178925 |
|  |  | *Camo-G2* | 178110 | 180829 |
|  |  | *HLA complex group 4 (HCG4), non-coding RNA* | 183413 | 185447 |
|  |  | *Camo-G3* | 184615 | 187359 |
|  |  | *HLA complex group 8 (HCG8), non-coding RNA* | 190147 | 191943 |
|  |  | *ZNRD1 antisense RNA 1 (ZNRD1-AS1), non-coding RNA* | 216873 | 218516 |
|  |  | *HLA complex group 4 (HCG4), non-coding RNA* | 230249 | 232330 |
|  |  | *Camo-G4* | 231498 | 234251 |
|  |  | *HLA complex group 8 (HCG8), non-coding RNA* | 237069 | 237632 |
|  |  | *HLA complex group 8 (HCG8), non-coding RNA* | 238787 | 239189 |

^a^ Nucleotide positions for *A. geoffroyi* and *C. moloch* sequences are on the consensus of overlapping BAC clones. For these two species, the genes contained in the overlapping BAC clones are in bold type.
