# Supplementary material for: Patterns of MHC-G-Like and MHC-B Diversification in New World Monkeys
Source: PLoS One. 2015 Jun 29;10(6):e0131343. doi: 10.1371/journal.pone.0131343 (PMC4486459; doi:10.1371/journal.pone.0131343)
Supplement: S2 Table — (DOCX) [file pone.0131343.s003.docx]

Table S2. Variability of MHC-G-like and –B positions predicted to interact with KIR domains D1 and D2 in *C. albifrons.*

| KIR Domain | MHC-I position | Variability within MHC-I clades | | | | |
| --- | --- | --- | --- | --- | --- | --- |
|  |  | G-Id | G-Ii | B-I | B-III | B-VI |
| D1 | 69 | D, A | A | D, A | D | A |
|  | 72 | Q, E | Q | Q, K | Q | Q |
|  | 75 | R | R | R | R | R |
|  | 76 | V | V | V | V | V |
|  | 79 | R | R | R, L | R | R |
|  | 80 | N, T | I | T | T | D |
|  | 83 | G, S | G | G, C | S | S, G |
|  | 84 | Y | Y | Y | C | S |
| D2 | 142 | I, N | I | I, N | I | V |
|  | 145 | R | R | R | R | R, G |
|  | 146 | K, N, M | K | K | Q | M, K |
|  | 149 | A, T | A | A | A | A |
|  | 150 | A, G | A | A | N | D, E |
|  | 151 | N | N | N | N | K |
