# Supplementary material for: Patterns of MHC-G-Like and MHC-B Diversification in New World Monkeys
Source: PLoS One. 2015 Jun 29;10(6):e0131343. doi: 10.1371/journal.pone.0131343 (PMC4486459; doi:10.1371/journal.pone.0131343)
Supplement: S3 Table — (DOCX) [file pone.0131343.s004.docx]

S3 Table. Published Platyrrhini MHC class I and KIR sequences used for phylogenetics and Interaction analyses.

| ***Name*** | ***Description*** | ***Accession*** |
| --- | --- | --- |
| Aona-g10303 | Aotus nancymaae clone Aona-g10303 | AY659835.1 |
| Aona-g20108 | Aotus nancymaae clone Aona-g20108 | AY659838.1 |
| Aona-g60101 | Aotus nancymaae clone Aona-g60101 | AY659849.1 |
| Aotr-G01 | Aotus trivirgatus MHC class I (Aotr-G*01) | U59644.1 |
| Aotr-G02 | Aotus trivirgatus MHC class I (Aotr-G*02) | U59645.1 |
| Aotr-G03 | Aotus trivirgatus MHC class I (Aotr-G*03) | U59646.1 |
| Aotr-PS2 | Aotus trivirgatus MHC class I processed pseudogene-2 | U52114.1 |
| Aotr-B1 | Aotus trivirgatusAotr-B1 gene for MHC class I antigen | AB113204.1 |
| Aotr-B2 | Aotus trivirgatusAotr-B2 gene for MHC class I antigen | AB113205.1 |
| Aotr-F | Aotus trivirgatusAotr-F gene for MHC class I antigen | AB113203.1 |
| Aotr-G2 | Aotus trivirgatusAotr-G2 gene for MHC class I antigen | AB113202.1 |
| Aovo-g10301 | Aotus vociferans clone Aovo-g10301 | AY659829.1 |
| Aovo-g20108 | Aotus vociferans clone Aovo-g20108 | AY659830.1 |
| Aovo-g60101 | Aotus vociferans clone Aovo-g60101 | AY659832.1 |
| Atbe-B1*01 | Ateles belzebuth MHC class I (Atbe-B01) | U59648.1 |
| Atbe-G*01 | Ateles belzebuth MHC class I (Atbe-G*01) | U59647.1 |
| Atbe-G*02 | Ateles belzebuth MHC class I (Atbe-G*02) | U59649.1 |
| Atbe-G*03 | Ateles belzebuth MHC class I (Atbe-G*03) | U59650.1 |
| AtfuA1-23 | Ateles fusciceps isolate A1-23 MHC class I antigen A | KC506567.1 |
| AtfuA2-1 | Ateles fusciceps isolate A2-1 MHC class I antigen A | KC506569.1 |
| AtfuA2-11 | Ateles fusciceps isolate A2-11 MHC class I antigen A | KC506571.1 |
| AtfuB1-4 | Ateles fusciceps isolate B1-4 MHC class I antigen B | KC506578.1 |
| AtfuB2-16 | Ateles fusciceps isolate B2-16 MHC class I antigen B | KC506589.1 |
| AtfuB2-3 | Ateles fusciceps isolate B2-3 MHC class I antigen B | KC506587.1 |
| AtfuB2-35 | Ateles fusciceps isolate B2-35 MHC class I antigen B | KC506586.1 |
| AtfuB2-5 | Ateles fusciceps isolate B2-5 MHC class I antigen B | KC506575.1 |
| Caja-B2 | Callithrix jacchus Caja-B2 pseudogene for mhc I B2 | AB636348.1 |
| Caja-B3 | Callithrix jacchus Caja-B3 gene for mhc I B3 | AB636349.1 |
| Caja-B4 | Callithrix jacchus Caja-B4 gene for mhc I B4 | AB636350.1 |
| Caja-B5 | Callithrix jacchus Caja-B5 pseudogene for mhc I B5 | AB636351.1 |
| Caja-B6 | Callithrix jacchus Caja-B6 gene for mhc class I B6 | AB636352.1 |
| Caja-B7 | Callithrix jacchus Caja-B7 gene for mhc class I B7 | AB636353.1 |
| Caja-B9 | Callithrix jacchus Caja-B9 pseudogene for mhc class I B9 | AB636355.1 |
| CajaIB6-47 | Callithrix jacchus isolate IB6-47 | KC510693.1 |
| CajaIB7-31 | Callithrix jacchus isolate IB7-31 | KC510695.1 |
| Caja-G01 | Callithrix jacchus MHC class I (Caja-G*01) | U59637.1 |
| Caja-G*02 | Callithrix jacchus MHC class I (Caja-G*02) | U59638.1 |
| Caja-G*04 | Callithrix jacchus MHC class I (Caja-G*04) | U59640.1 |
| Caja-Ga15 | Callithrix jacchus MHC class I antigen (Caja-G) Caja-G*a15 | JX826616.1 |
| Caja-G*06 | Callithrix jacchus for MHC class I antigen Caja-G*06 | HE962236.1 |
| Caja-G03 | Callithrix jaccus MHC class I (Caja-G*03) | U59639.1 |
| Caja-G05 | Callithrix jaccus MHC class I (Caja-G*05) | U59641.1 |
| Caja-PS2 | Callithrix jaccus MHC class I processed pseudogene-2 | U52115.1 |
| Pipi-B01 | Pithecia pithecia MHC class I (Pipi-B01) | U59654.1 |
| Pipi-G*01 | Pithecia pithecia MHC class I (Pipi-G*01) | U59651.1 |
| Pipi-G*02 | Pithecia pithecia MHC class I (Pipi-G*02) | U59652.1 |
| Pipi-G*03 | Pithecia pithecia MHC class I (Pipi-G*03) | U59653.1 |
| Pipi-G*04 | Pithecia pithecia MHC class I (Pipi-G*04) | U59655.1 |
| Pipi-G*05 | Pithecia pithecia MHC class I (Pipi-G*05) | U59656.1 |
| Safu-G04) | Saguinus fuscicollis MHC class I (Safu-G*04) | U59636.1 |
| Sage-PS1 | Saguinus geoffroyi MHC class I processed pseudogene-1 | U52118.1 |
| Sage-PS2 | Saguinus geoffroyi MHC class I Prossesed Pseudogene-2 | U52119.1 |
| Sala-G0306 | Saguinus labiatus MHC class I antigen G Sala-G*03:06 | JF785561.1 |
| Samy-PS2 | Saguinus mystax MHC class I processed pseudogene-2 | U52121.1 |
| Saoe-G12 | Saguinus oedipus MHC class I antigen Saoe-G pseudogene | AF020686.1 |
| Saoe-G13 | Saguinus oedipus MHC class I antigen Saoe-G pseudogene | AF020687.1 |
| Saoe-G14 | Saguinus oedipus MHC class I antigen Saoe-G pseudogene | AF020688.1 |
| Saoe-G15 | Saguinus oedipus MHC class I antigen Saoe-G pseudogene | AF020689.1 |
| Saoe-G16 | Saguinus oedipus MHC class I antigen Saoe-G pseudogene | AF020690.1 |
| Saoe-PS1 | Saguinus oedipus MHC class I processed pseudogene-1 | U52113.1 |
| Sasc-G01 | Saimiri sciureus MHC class I (Sasc-G*01) | U59657.1 |
| Sasc-G02 | Saimiri sciureus MHC class I (Sasc-G*02) | U59658.1 |
| Sasc-G03 | Saimiri sciureus sciureus MHC class I antigen (Sasc-G) | AY282760.1 |
| Sasc-G04 | Saimiri sciureus sciureus MHC class I antigen (Sasc-G) | AY282761.1 |
| Sasc-G05 | Saimiri sciureus sciureus MHC class I antigen (Sasc-G) | AY282762.1 |
| Sasc25 | Saimiri sciureus sciureus Sasc-25 gene for MHC class I | AJ438577.1 |
| Sasc31 | Saimiri sciureus sciureus Sasc-31 gene for MHC class I | AJ438576.1 |
|  |  |  |
| Lala KIR3DS3*01 | Lagothrix lagotricha killer-cell Ig-like receptor (KIR3DS3) mRNA | KF011974.1 |
| Lala KIR3DS1*02 | Lagothrix lagotricha killer-cell Ig-like receptor (KIR3DS1) mRNA | KF011972.1 |
| Lala KIR3DL2*04 | Lagothrix lagotricha killer-cell Ig-like receptor-like (KIR3DL2) | KF011969.1 |
| Lala KIR3DL1*03 | Lagothrix lagotricha killer-cell Ig-like receptor-like (KIR3DL1) | KF011965.1 |
| SaboKIR1 | Saimiri boliviensis killer cell immunoglobulin-like receptor 3DL2 | XM_010331950.1 |
| SaboKIR4 | Saimiri boliviensis killer cell immunoglobulin-like receptor 3DL1 | XM_003944071.2 |
| SaboKIR7 | Saimiri boliviensis killer cell immunoglobulin-like receptor 3DL1 | XM_003945045.2 |
| SaboKIR9 | Saimiri boliviensis killer cell immunoglobulin-like receptor 3DL2 | XM_010331983.1 |
| Atbe KIR3DS3*01 | Ateles belzebuth killer-cell Ig-like receptor (KIR3DS3) mRNA | KF011962.1 |
| Atbe KIR2DS3 | Ateles belzebuth killer-cell Ig-like receptor-like (KIR2DS3) mRNA | KF011960.1 |
| Atbe KIR3DL2 | Ateles belzebuth killer-cell Ig-like receptor-like (KIR3DL2) mRNA | KF011958.1 |
